# Relationship between duration and extent of oedema and visual acuity outcome with ranibizumab in diabetic macular oedema: A post hoc analysis of Protocol I data

**DOI:** 10.1038/s41433-019-0522-z

**Published:** 2019-07-18

**Authors:** Srinivas R. Sadda, Joanna Campbell, Pravin U. Dugel, Nancy M. Holekamp, Szilárd Kiss, Anat Loewenstein, Albert J. Augustin, Vanessa Shih, Xiaoshu Xu, Charles C. Wykoff, Scott M. Whitcup

**Affiliations:** 1 Doheny Eye Institute, Los Angeles, CA USA; 2 Department of Ophthalmology, David Geffen School of Medicine at UCLA, Los Angeles, CA USA; 3 Allergan plc, Irvine, CA USA; 4 Retinal Consultants of Arizona, Phoenix, AZ USA; 5 Department of Ophthalmology, Keck School of Medicine, University of Southern California, Los Angeles, CA USA; 6 Pepose Vision Institute and Washington University School of Medicine, St. Louis, MO USA; 7 Weill Cornell Medical College, New York, NY USA; 8 Department of Ophthalmology, Tel Aviv Medical Center and Sackler Faculty of Medicine, Tel Aviv University, Tel Aviv, Israel; 9 Department of Ophthalmology, Staedtisches Klinikum Karlsruhe, Karlsruhe, Germany; 10 Retina Consultants of Houston, Blanton Eye Institute, Houston Methodist Hospital, Houston, TX USA; 11 Jules Stein Eye Institute, David Geffen School of Medicine at UCLA, Los Angeles, CA USA

**Keywords:** Retinal diseases, Visual system

## Abstract

**Background/objectives:**

This post hoc analysis explores the relationship between residual oedema exposure after ranibizumab treatment initiation and long-term visual acuity outcome in eyes with centre-involved diabetic macular oedema (DMO).

**Subjects/methods:**

Eyes randomised to the ranibizumab + prompt or deferred laser treatment arms in the Protocol I trial and with observed central retinal thickness (CRT) readings at baseline and ≥1 follow-up visits (*n* = 367) were stratified by 1) oedema duration (number of study visits with CRT ≥ 250 µm during the first 52 weeks of ranibizumab treatment); and 2) oedema extent (amount of excess CRT [≥ 250 µm] at each study visit, averaged over the first 52 weeks). Associations between measures of residual oedema and best-corrected visual acuity (BCVA) were assessed in multiple regression analyses.

**Results:**

Oedema duration and oedema extent during the first 52 weeks of ranibizumab treatment showed significant negative associations with BCVA improvement at weeks 52, 104 and 156. Eyes with the most persistent oedema gained (mean) 4.4 (95% CI 0.1─8.7) fewer Early Treatment Diabetic Retinopathy Study (ETDRS) letters at week 156 than eyes with the least persistent oedema (*P* *=* 0.044). Eyes with the greatest amount of oedema gained (mean) 9.3 (95% CI 4.0─14.5) fewer ETDRS letters at week 156 than eyes with the least amount of oedema (*P* < 0.001).

**Conclusions:**

Macular oedema exposure over the first 52 weeks of ranibizumab treatment is a negative prognostic factor for long-term visual acuity improvement in centre-involved DMO.

## Introduction

Intravitreal vascular endothelial growth factor-A antagonists (anti-VEGF agents) are currently regarded as appropriate first-line therapy for most patients with centre-involved diabetic macular oedema (DMO), resulting in visual acuity loss [1]. Randomised clinical trials indicate that these agents are more effective than laser photocoagulation in reducing macular thickness and improving visual acuity in eyes with centre-involved DMO [2–4]. However, despite the intensive treatment schedules employed in clinical trials, anatomic and visual responses to anti-VEGF therapy are often incomplete, with ~20–65% of eyes demonstrating persistent retinal thickening [2, 5–7] and ~30–65% of eyes failing to achieve ≥10-letter improvement in best-corrected visual acuity (BCVA) [2, 3, 5, 8–11] after 1 or 2 years of treatment.

The reasons for inter-subject variation in response to anti-VEGF therapy in DMO are incompletely understood, with polymorphisms in the *VEGF* gene [12], differences in the *VEGF* gene expression [13], disease phenotype [14], glycaemic control [15], macular ischaemia [16], oedema chronicity [17, 18], subfoveal choroidal thickness [19] and foveal atrophy [17], variously implicated as possible contributory factors. A post hoc analysis indicating relatively limited visual acuity improvement in eyes receiving deferred as opposed to prompt ranibizumab treatment in the RISE and RIDE clinical trials suggests that chronic macular oedema might reduce the capacity for vision gain in DMO [20]. The disruption of retinal architecture that accompanies DMO [21] may result in retinal glial proliferation, neuronal cell loss and compromised visual function, persisting in some cases even after oedema resolution [22–26]. The published literature is, however, inconsistent regarding the relationship between the anatomic and functional responses to laser photocoagulation [27], corticosteroid [14] and anti-VEGF [18, 28, 29] therapy in DMO.

Early identification of patients who are unlikely to benefit from continuation of anti-VEGF therapy would allow alternative, potentially more effective, disease management strategies to be considered at an earlier stage in the disease process. The Early Anti-VEGF Response and Long-term Efficacy programme, a series of post hoc analyses of data from the Diabetic Retinopathy Clinical Research Network’s Protocol I trial [5], one of the largest published studies of ranibizumab in DMO, was initiated to explore the relationship between early and long-term anti-VEGF treatment outcomes. Analyses to date have demonstrated a significant association between early (12-week) and long-term (1- to 3-year) BCVA improvement with ranibizumab [30], but an apparent dissociation between early reduction in macular thickness and long-term BCVA improvement [31]. Given that the use of anti-VEGF treatment in clinical practice is based in large part on optical coherence tomography changes, the present analysis explores the anatomic–functional relationship further by assessing macular thickness over the course of ranibizumab treatment rather than at a single time point.

## Methods

### Protocol I study—an overview

Protocol I was a prospective Phase III randomised clinical trial (ClinicalTrials.gov identifier NCT00445003) that compared intravitreal ranibizumab 0.5 mg + prompt (within 3–10 days) or deferred (after ≥24 weeks) laser versus sham intravitreal injection + prompt laser versus intravitreal triamcinolone 4 mg + prompt laser in 691 patients (854 study eyes) with centre-involved DMO. The study protocol was approved by multiple institutional review boards, and all patients provided their written informed consent prior to study participation. The study methodology is detailed elsewhere [5]. For study inclusion, patients were required to have a baseline BCVA of 78 to 24 Early Treatment Diabetic Retinopathy Study (ETDRS) letters (approximate Snellen equivalent 20/32‒20/320), and central subfield retinal thickness (CRT) of ≥250 µm, as determined by time-domain OCT (Stratus, Carl Zeiss Meditec Inc., Dublin, CA, USA). Intravitreal injections were performed every 4 weeks for the first 12 weeks, and as needed thereafter; the frequency of laser retreatment was governed by the extent of central macular oedema. BCVA and CRT measurements were performed every 4 weeks for the first 52 weeks and every 4–16 weeks thereafter. Study findings at 1 and 2 years demonstrated an efficacy advantage in the ranibizumab treatment arms [5, 32], and patients in the sham injection and intravitreal triamcinolone treatment arms were offered the option of switching to open-label ranibizumab treatment for the third year [33]. Follow-up findings for eyes randomised to ranibizumab plus prompt or deferred laser treatment indicated that the vision gains achieved during the first 2 years of treatment were largely maintained at 3 [33] and 5 years [34].

### Anatomic and visual response analysis

This analysis is based on 3-year follow-up data from Protocol I study eyes that were randomised to ranibizumab plus prompt or deferred laser treatment, and in addition, provided an observed CRT reading at baseline and at ≥1 follow-up visits. Pooled study eyes from the two ranibizumab treatment arms were independently stratified according to 1) *duration* of oedema following initiation of ranibizumab, as defined by the cumulative number of study visits (sequential or nonsequential) with CRT ≥250 µm during the first 52 weeks of treatment; and 2) *extent* of oedema following initiation of ranibizumab, as defined by the amount of oedema (CRT ≥250 µm) at each study visit, averaged over the first 52 weeks of treatment. The amount of oedema at each visit was quantified as the observed CRT minus 250 μm for eyes with CRT ≥250 μm, and as 0 μm for eyes with CRT < 250 μm. Based on their stratification by duration of oedema, study eyes were categorised as Cohort 1: 0–3 visits, Cohort 2: 4–7 visits, Cohort 3: 8–11 visits and Cohort 4: 12–14 visits over the first 52 weeks (study visits were scheduled every 4 weeks for the first 52 weeks of ranibizumab treatment; accordingly, the maximum possible number of visits during this period was 14). Independently, based on their stratification by extent of oedema, study eyes were categorised as Quartile 1: percentiles 0–25, Quartile 2: percentiles 26–50, Quartile 3: percentiles 51–75 and Quartile 4: percentiles 76–100 of the average amount of oedema over the first 52 weeks.

### Persistence of macular oedema into the second year of ranibizumab treatment

Persistence of macular oedema into the second year of ranibizumab treatment was assessed in terms of 1) the cumulative number of study visits (sequential or nonsequential) with CRT ≥250 µm during weeks 52–104 (for cohorts categorised according to the duration of oedema over the first 52 weeks of treatment), and 2) the average amount of oedema (CRT ≥250 µm) during weeks 52–104 (for quartiles categorised according to the average extent of oedema over the first 52 weeks of treatment).

### Relationship between duration and extent of oedema and visual acuity outcome

To evaluate the relationship between oedema duration/extent during the first 52 weeks of ranibizumab therapy and long-term visual acuity outcome, BCVA responses (mean change from baseline in BCVA; proportion of study eyes with BCVA improvement ≥10 ETDRS letters) at weeks 52, 104, and 156 were compared across Cohorts 1–4 (duration) and Quartiles 1–4 (extent). Any missing CRT and BCVA readings at the relevant time points—whether due to missed or omitted follow-up visits—were imputed using the last-observation-carried-forward method. Multiple linear and logistic regression models incorporating covariates for baseline characteristics (age, baseline BCVA, baseline CRT and prior DMO treatment [Yes/No]) and treatment intensity (cumulative number of ranibizumab injections and laser procedures) were used to explore the association between oedema duration/extent (the primary explanatory covariate) and long-term BCVA response.

### Statistical analysis

Baseline characteristics and visual acuity outcomes were compared across oedema categories using KruskalWallis one-way analysis of variance (continuous variables) and Pearson"s chi-square test (categorical variables). Pairwise comparisons were performed with the Student’s *t*-test (continuous variables) and Pearson’s chi-square test (categorical variables). Correlations between oedema duration/extent in years 1 and 2 were assessed using Pearson’s correlation. Probability values in the linear and logistic regression analyses were determined using Student’s *t*-test. Statistical analyses were performed with SAS versions 9.3 and 9.4 (SAS Inc., Cary, NC, USA). A *P* value of <0.05 was considered statistically significant.

## Results

In total, 375 eyes were assigned to the ranibizumab + prompt or deferred laser treatment arms in the Protocol I trial; of these, 367 eyes were eligible for inclusion in this analysis.

### Duration of oedema analysis

Of the analysis population, 23.2% of eyes (*n* = 85) had 0–3 study visits, 24.5% (*n* = 90) had 4–7 study visits, 19.1% (*n* = 70) had 8–11 study visits and 33.2% (*n* = 122) had 12–14 study visits with CRT ≥ 250 µm during the first 52 weeks of ranibizumab treatment (Cohorts 1, 2, 3 and 4, respectively).

#### Persistence of macular oedema into year 2 of treatment

The pattern of study visits with macular thickening during the first year of ranibizumab treatment was largely replicated during the second year. Eyes with the most persistent (Cohort 4) and least persistent (Cohort 1) macular oedema during the first 52 weeks generally displayed the most persistent and least persistent oedema, respectively, during the second 52 weeks. The cumulative number of study visits with CRT ≥ 250 µm in the first and second 52-week periods showed a significant correlation (Pearson correlation coefficient = 0.742; *P* *<* 0.001).

#### Relationship between duration of oedema and long-term visual acuity outcome

**Fig. 1:**
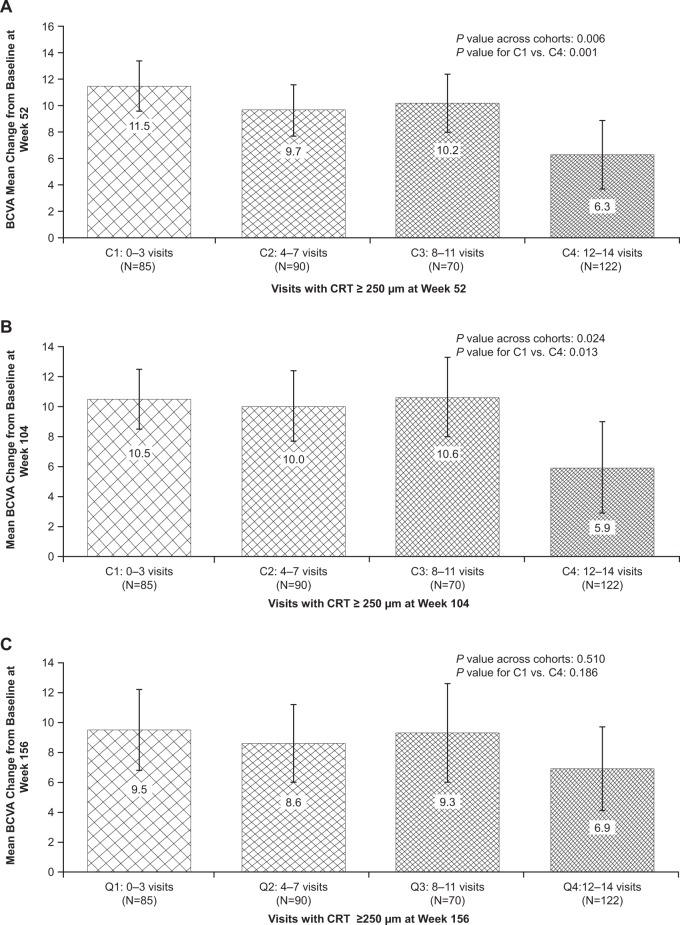
Mean (95% CI) change from baseline in BCVA at **a** week 52, **b** week 104 and **c** week 156 among study eyes categorised by the cumulative number of study visits with CRT ≥ 250 µm during the first 52 weeks of ranibizumab treatment (*N* = 367). *BCVA* best-corrected visual acuity,* C* cohort, *CRT* central subfield retinal thickness, *Q* quartile

In unadjusted analyses, eyes with more persistent macular oedema during the first 52 weeks of ranibizumab treatment showed less long-term BCVA improvement than eyes with less persistent macular oedema. The mean (± standard deviation [SD]) BCVA improvement from baseline (Cohort 4 versus Cohort 1) was 6.3 (± 14.3) versus 11.5 (± 8.9) ETDRS letters (*P* *=* 0.001) at week 52, 5.9 (± 17.0) versus 10.5 (± 9.3) ETDRS letters (*P* *=* 0.013) at week 104 and 6.9 (± 15.6) versus 9.5 (± 12.7) ETDRS letters (*P* *=* 0.186) at week 156 (Fig. 1).

Inter-cohort comparisons revealed significant differences in baseline CRT, prior DMO treatment and on-study treatment intensity between eyes with more persistent versus less persistent macular oedema during the first 52 weeks of ranibizumab therapy (Table 1). After adjusting for age, baseline BCVA and CRT, prior DMO treatment and the cumulative number of on-study laser procedures and ranibizumab injections (‘the standard covariates’), multiple linear regression analysis demonstrated a significant negative association between oedema duration during the first 52 weeks and BCVA improvement (from baseline) at 52, 104 and 156 weeks. Compared with Cohort 1 (least persistent macular oedema), Cohort 4 (most persistent oedema) was estimated to have gained, on average, 4.4 (95% confidence interval [CI] 1.0–7.9) fewer ETDRS letters at week 52 (*P* *=* 0.012), 6.2 (95% CI 2.1–10.2) fewer ETDRS letters at week 104 (*P* *=* 0.003) and 4.4 (95% CI 0.1–8.7) fewer ETDRS letters at week 156 (*P* *=* 0.044) (Table 2).

**Table 1:** Baseline characteristics and treatment intensity of study eyes categorised by duration of oedema (cumulative number of study visits with CRT ≥ 250 µm) during the first 52 weeks of ranibizumab treatment (*N* = 367)

Characteristic	Cumulative number of visits with CRT ≥250 µm during the first 52 weeks				
	Cohort 1 ≤ 3 visits (*n* = 85)	Cohort 2 4–7 visits (*n* = 90)	Cohort 3 8–11 visits (*n* = 70)	Cohort 4 12–14 visits (*n* = 122)	*P* value^a^
Mean age, years	61.3	62.6	63.9	63.3	0.451
Male, *n* (%)	40 (47.1)	51 (56.7)	42 (60.0)	74 (60.7)	0.232
Mean baseline BCVA, ETDRS letters	64.7	62.4	60.4	63.1	0.223
Mean baseline CRT, µm	339	415	420	439	<0.001
Prior DMO therapy, *n* (%)	41 (48.2)	57 (63.3)	48 (68.6)	77 (63.1)	0.048
Cumulative no. of ranibizumab injections at week 52, mean	5.8	8.0	9.2	10.1	<0.001
Cumulative no. of ranibizumab injections at week 104, mean	6.6	10.9	12.6	14.2	<0.001
Cumulative no. of ranibizumab injections at week 156, mean	7.1	12.9	15.4	16.8	<0.001
Cumulative no. of laser procedures at week 52, mean	0.8	1.1	1.4	1.7	<0.001
Cumulative no. of laser procedures at week 104, mean	0.9	1.5	2.0	2.3	<0.001
Cumulative no. of laser procedures at week 156, mean	1.1	1.7	2.1	2.6	<0.001

*BCVA* best-corrected visual acuity, *CRT* central subfield retinal thickness, *DMO* diabetic macular oedema, *ETDRS* Early Treatment Diabetic Retinopathy Study

^a^Comparison across all four cohorts, using Kruskal–Wallis one-way analysis of variance for continuous variables and Pearson’s chi-square test for categorical variables

**Table 2:** Estimated differences in long-term BCVA outcomes among study eyes categorised by 1) duration of oedema (cumulative number of study visits with CRT ≥250 µm) and 2) average extent of oedema (CRT ≥250 µm) over the first 52 weeks of ranibizumab treatment, after adjustment for potential confounders

Time	Estimated difference (95% CI) in BCVA improvement (ETDRS letters)^a^	*P* value	Odds ratio (95% CI) of achieving ≥10-letter BCVA improvement^a^	*P* value
Study eyes categorised by duration of oedema (cumulative number of study visits with CRT ≥250 µm) over the first 52 weeks of ranibizumab treatment				
**Week 52**				
Cohort 2	−1.9 (−5.1 to 1.4)	0.253	0.58 (0.29–1.16)	0.124
Cohort 3	−1.3 (−4.9 to 2.2)	0.461	0.45 (0.21–0.99)	0.047
Cohort 4	−4.4 (−7.9 to −1.0)	0.012	0.31 (0.15–0.67)	0.003
**Week 104**				
Cohort 2	−2.1 (−5.9 to 1.8)	0.289	0.47 (0.23–0.94)	0.032
Cohort 3	−1.7 (−6.0 to 2.5)	0.417	0.45 (0.21–0.96)	0.040
Cohort 4	−6.2 (−10.2 to −2.1)	0.003	0.23 (0.11–0.50)	<0.001
**Week 156**				
Cohort 2	−2.6 (−6.7 to 1.5)	0.216	0.37 (0.18–0.74)	0.005
Cohort 3	−2.5 (−7.1 to 2.1)	0.280	0.41 (0.19–0.89)	0.024
Cohort 4	−4.4 (−8.7 to −0.1)	0.044	0.31 (0.15–0.63)	0.001
Study eyes categorised by average amount of excess oedema (CRT > 250 μm) over the first 52 weeks of ranibizumab treatment				
**Week 52**				
Quartile 2	−1.7 (−4.8 to 1.5)	0.297	0.51 (0.26–1.00)	0.051
Quartile 3	−5.3 (−8.9 to −1.8)	0.003	0.39 (0.18–0.84)	0.016
Quartile 4	−6.9 (−11.1 to −2.7)	0.001	0.28 (0.11–0.71)	0.007
**Week 104**				
Quartile 2	−3.1 (−6.8 to 0.7)	0.107	0.42 (0.21–0.83)	0.012
Quartile 3	−6.0 (−10.1 to −1.9)	0.005	0.28 (0.13–0.61)	0.001
Quartile 4	−9.8 (−14.7 to −4.8)	<0.001	0.21 (0.08–0.54)	0.001
**Week 156**				
Quartile 2	−3.6 (−7.6 to 0.4)	0.075	0.40 (0.20–0.78)	0.007
Quartile 3	−7.0 (−11.4 to −2.6)	0.002	0.21 (0.10–0.46)	<0.001
Quartile 4	−9.3 (−14.5 to −4.0)	<0.001	0.13 (0.05–0.34)	<0.001

*BCVA* best-corrected visual acuity, *CI* confidence interval, *CRT* central retinal thickness, *DMO* diabetic macular oedema, *ETDRS* Early Treatment Diabetic Retinopathy Study

^a^Estimated differences and odds ratios are expressed relative to Cohort/Quartile 1, with adjustment for age, baseline BCVA, baseline CRT, prior DMO treatment and cumulative number of ranibizumab injections and laser procedures

Multiple logistic regression analysis with adjustment for the standard covariates indicated a significant negative association between duration of oedema during the first 52 weeks of ranibizumab treatment and categorical BCVA response (proportion of eyes with ≥10-letter improvement) at 52, 104 and 156 weeks. Compared with Cohort 1, eyes in the two categories with the most persistent macular oedema (Cohorts 3 and 4) were significantly less likely to achieve ≥10-letter improvement in BCVA at weeks 52, 104, and 156 (Table 2).

### Extent of oedema analysis

Among individual study eyes, the average amount of oedema (based on a CRT threshold of 250 μm) over the first 52 weeks of ranibizumab treatment ranged from 0 to 287 μm. Stratification of eyes by percentile of the average amount of oedema yielded the following cohorts: Quartile 1: average excess CRT 0–9 μm (mean average 4 μm) (*n* = 92); Quartile 2: average excess CRT 9–29 μm (mean average 18 μm) (*n* = 92); Quartile 3: average excess CRT 29–74 μm (mean average 47 μm) (*n* = 92); Quartile 4: average excess CRT 75–287 μm (mean average 129 μm) (*n* = 91).

#### Persistence of macular oedema into year 2 of treatment

The pattern of distribution of oedema between study eyes during the first year of ranibizumab treatment was generally maintained during the second year. Eyes having the highest (Quartile 4) and lowest (Quartile 1) average amount of oedema during the first 52 weeks of ranibizumab treatment predominantly showed the highest and lowest average amounts, respectively, of oedema during the second 52 weeks. The average amount of oedema was significantly correlated between the first and second 52-week periods (Pearson correlation coefficient = 0.673; *P* *<* 0.001).

#### Relationship between oedema severity and long-term visual acuity outcome

**Fig. 2:**
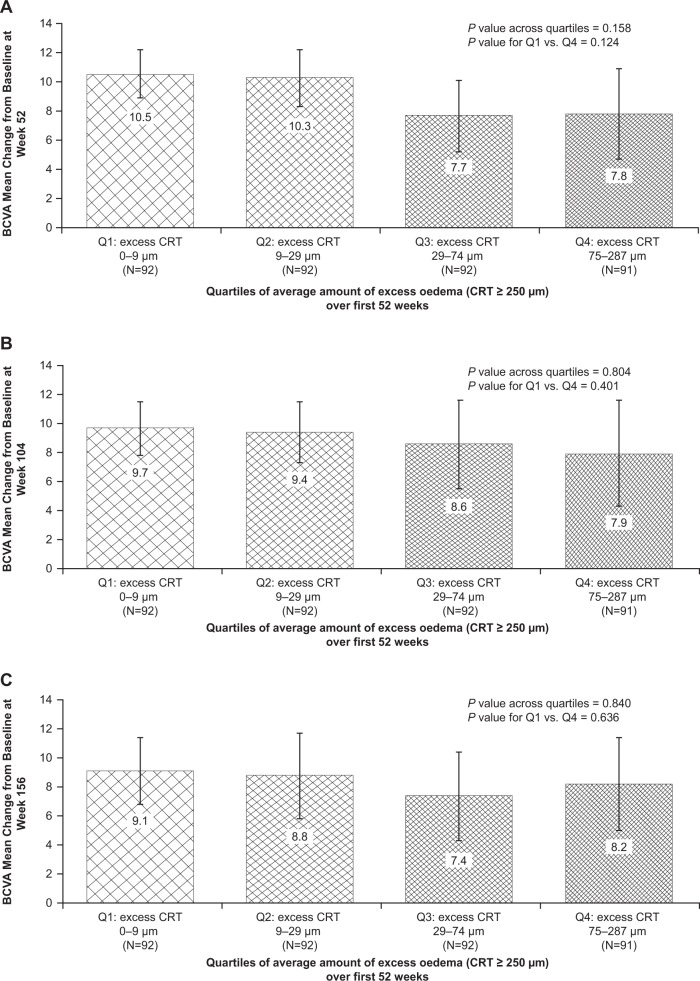
Mean (95% CI) change from baseline in BCVA at **a** week 52, **b** week 104 and **c** week 156 among study eyes categorised by the average amount of excess oedema (CRT ≥250 µm) during the first 52 weeks of ranibizumab treatment (*N* = 367). *BCVA* best-corrected visual acuity, *CRT* central subfield retinal thickness, *Q* quartile

Unadjusted analyses indicated a tendency for eyes with higher levels of oedema during the first 52 weeks of ranibizumab treatment to show less pronounced long-term BCVA improvement than eyes with lower levels of oedema. The mean (±SD) BCVA improvement from baseline (Quartile 4 vs. Quartile 1) was 7.8 (±14.7) versus 10.5 (±8.1) ETDRS letters (*P* *=* 0.124) at week 52, 7.9 (±17.4) versus 9.7 (±8.8) ETDRS letters (*P* *=* 0.401) at week 104 and 8.2 (±15.2) versus 9.1 (±11.1) ETDRS letters (*P* *=* 0.636) at week 156 (Fig. 2).

Significant differences in baseline BCVA and CRT, and on-study treatment intensity were noted between eyes with higher and lower average amounts of macular oedema during the first 52 weeks of ranibizumab therapy (Table 3). Multiple linear regression analysis with adjustment for the standard covariates demonstrated a significant negative association between average amount of macular oedema during the first 52 weeks of ranibizumab treatment and BCVA improvement at 52, 104 and 156 weeks. Compared with eyes with the lowest average amount of oedema (Quartile 1), eyes with the highest amount of oedema (Quartile 4) were estimated to have gained, on average, 6.9 (95% CI 2.7–11.1) fewer ETDRS letters at week 52 (*P* *=* 0.001), 9.8 (95% CI 4.8–14.7) fewer ETDRS letters at week 104 (*P* *<* 0.001) and 9.3 (95% CI 4.0–14.5) fewer ETDRS letters at week 156 (*P* *<* 0.001) (Table 2).

**Table 3:** Baseline characteristics and treatment intensity of study eyes categorised by extent of oedema (average amount of oedema [CRT ≥250 µm]) during the first 52 weeks of ranibizumab treatment (*N* = 367)

Characteristic	Average amount of oedema (CRT ≥250 µm) during the first 52 weeks				
	Quartile 1 0–9 µm (*n* = 92)	Quartile 2 9–29 µm (*n* = 92)	Quartile 3 29–74 µm (*n* = 92)	Quartile 4 75–287 µm (*n* = 91)	*P* value^a^
Mean age, years	62.0	63.1	63.7	62.4	0.660
Male, *n* (%)	46 (50.0)	50 (54.3)	54 (58.7)	57 (62.6)	0.344
Mean baseline BCVA, ETDRS letters	66.6	62.9	61.9	59.7	0.001
Mean baseline CRT, µm	307	380	433	506	<0.001
Prior DMO therapy, *n* (%)	48 (52.2)	58 (63.0)	58 (63.0)	59 (64.8)	0.275
Cumulative no. of ranibizumab injections at week 52, mean	6.0	7.9	9.3	10.5	<0.001
Cumulative no. of ranibizumab injections at week 104, mean	7.0	10.3	12.5	15.7	<0.001
Cumulative no. of ranibizumab injections at week 156, mean	7.8	11.8	14.9	18.9	<0.001
Cumulative no. of laser procedures at week 52, mean	0.9	1.3	1.3	1.7	0.002
Cumulative no. of laser procedures at week 104, mean	1.0	1.8	1.8	2.3	<0.001
Cumulative no. of laser procedures at week 156, mean	1.2	1.9	2.0	2.7	<0.001

*BCVA* best-corrected visual acuity, *CRT* central subfield retinal thickness, *DMO* diabetic macular oedema, *ETDRS* Early Treatment Diabetic Retinopathy Study

^a^Comparison across all four cohorts, using Kruskal–Wallis one-way analysis of variance for continuous variables and Pearson’s chi-square test for categorical variables

A significant negative association was noted between the average amount of oedema during the first 52 weeks of ranibizumab treatment and the likelihood of achieving a ≥10-letter improvement in BCVA at 52, 104 and 156 weeks. Compared with Quartile 1, Quartiles 3 and 4 were significantly less likely to show a ≥10-letter improvement in BCVA at weeks 52, 104 and 156 (Table 2).

## Discussion

This analysis of the Protocol I study data suggests that approximately one-quarter of eyes receiving intravitreal ranibizumab for centre-involved DMO achieved near-permanent resolution of macular oedema (as signified by ≤3 of a total of 14 study visits with CRT ≥250 µm during the first 52 weeks of treatment). In contrast, for one-third of ranibizumab-treated eyes, macular oedema persisted uninterrupted throughout most of the first 52 weeks (as signified by 12–14 of 14 study visits with CRT ≥250 µm). Moreover, of those eyes that showed chronic persistent oedema during the first year of ranibizumab treatment, approximately two-thirds continued to experience uninterrupted or near-uninterrupted oedema during the second year. These findings are consistent with those of an earlier exploration of DMO evolution under long-term ranibizumab treatment, which was also based on a subgroup analysis of the Protocol I study data [18]. Among study eyes with persistent oedema after 6 months of ranibizumab treatment (*n* = 117), the cumulative probability of chronic persistent DMO was 81% at 12 months, 56% at 24 months, and 40% at 36 months [18]. Thus, even with a sustained, as-needed retreatment regimen and close patient follow-up, resolution of chronic oedema is a slow, gradual and often incomplete process.

Unadjusted analysis of the Protocol I study data suggests that long-term vision outcome with ranibizumab is more closely influenced by the duration (persistence) than by the severity of macular oedema during the initial 52 weeks of treatment. However, after adjusting for potential confounders, both oedema duration and oedema severity showed significant negative association with long-term (1- to 3-year) visual acuity outcomes. Eyes with the most persistent macular oedema over the first 52 weeks of treatment gained, on average, 4.4 fewer ETDRS letters at week 156 than eyes with the least persistent macular oedema. Likewise, eyes with the greatest average amount of macular oedema over the first 52 weeks of treatment gained 9.3 fewer ETDRS letters at week 156 than eyes with the least amount of macular oedema. Hence, the improvement in visual acuity obtained with long-term ranibizumab treatment appears to be negatively associated with the overall burden (intensity and persistence) of exposure to oedema.

A unique feature of the current analysis is that anatomic response to ranibizumab was assessed longitudinally over the first 52 weeks of treatment rather than at a single time point. This is in contrast to previous Early Anti-VEGF Response and Long-term Efficacy programme analyses, which typically assessed anatomic response to ranibizumab at week 12 of treatment (i.e., after the first three monthly intravitreal injections) and failed to demonstrate a significant association between early CRT reduction and long-term BCVA improvement [31]. Similarly, retrospective analyses of the RISE/RIDE study data suggest a dissociation between early anatomic response to ranibizumab after the third monthly intravitreal injection and long-term (2-year) visual acuity outcome in DMO [29]. Accordingly, reduction of oedema exposure (chronicity and/or severity) over the first 52 weeks of ranibizumab treatment would appear to be of greater prognostic importance for long-term visual acuity improvement than acute reduction of macular thickness. In support of this proposition, an independent post hoc analysis of Protocol I study data indicated that early (week 16) and sustained (weeks 32 and 52) CRT reduction (≥20%) was associated with a better visual acuity outcome at week 52 than early but unsustained CRT reduction [28]. Moreover, a subsequent exploratory analysis of the Protocol I study data indicated that the long-term (3-year) visual acuity response of eyes with chronic persistent oedema through 3 years was significantly worse than that of eyes with less persistent oedema [18].

The presence of chronic oedema may signify a transition from the acute inflammation and vascular dysfunction characteristic of early-stage DMO to the chronic inflammation and neuronal damage of later-stage disease [14]. It is suggested that as the pathophysiology of DMO evolves, so too may the retinal response to anti-VEGF treatment [14]. Persistent and/or recurrent macular oedema has been implicated as a possible contributory factor to poor visual acuity outcome in ranibizumab-treated eyes [17, 18, 20]. Consequently, delay in initiating anti-VEGF treatment may potentially reduce the scope for vision improvement [20]. Consistent with this hypothesis, a post hoc analysis of the RISE/RIDE studies reported that BCVA improvement in eyes randomised to 2 years of sham intravitreal injection and then switched to monthly ranibizumab 0.5 mg treatment for the third year (mean 2.8 ETDRS letters) was substantially less than that achieved after the first year of ranibizumab treatment in eyes randomised to monthly ranibizumab 0.3 or 0.5 mg from the outset (mean 10.6 and 11.1 ETDRS letters, respectively) [20]. Accordingly, retention of residual fluid on the macula would appear to have a detrimental effect over time, limiting the potential visual benefit that might be achieved with anti-VEGF therapy.

This analysis has a number of strengths, including its large sample size, extended treatment duration, and use of data based on a randomised study design, and a standardised retreatment protocol and OCT methodology. Although the axial resolution and retinal layer delineation provided by time-domain OCT scanning (the modality employed in the Protocol I study) are inferior to those of the spectral-domain OCT devices currently in use [35], time-domain OCT measurements are nevertheless reproducible and correlate with other morphometric parameters of macular anatomy in DMO [36]. Persistence of the association between oedema duration/intensity and visual outcome after raising the specified threshold for macular oedema to CRT ≥300 µm in a sensitivity analysis (results not shown) attests to the robustness of the study findings. Limitations of the analysis include its retrospective design, the imputation of missing data (last-observation-carried-forward method) and the absence of information on other factors that might influence DMO progression and response to anti-VEGF therapy, such as disease duration, subtype and morphologic pattern, severity of diabetic retinopathy and macular ischaemia and level of glycaemic control [28, 37–40].

In summary, the current analysis re-examines the relationship between anatomic and functional response to ranibizumab and demonstrates that greater exposure to macular oedema over the first year of treatment is associated with significantly worse visual acuity outcome. Oedema exposure, as measured by the duration of macular oedema or by the average amount of excess macular thickness over the first 52 weeks of treatment, was shown to be a negative prognostic factor for long-term (3-year) visual acuity improvement in ranibizumab-treated eyes with centre-involved DMO. Assessment of anatomic response over multiple time points suggests that the structure–function relationship revealed with ranibizumab in DMO is reasonably robust. For patients with persistent macular oedema during anti-VEGF therapy, further research is required to assess the benefits of early introduction of additional or alternative disease management strategies and their optimal timing. The recent DRCR.net Protocol U study, which evaluated the effect of adjunctive intravitreal dexamethasone in ranibizumab-treated eyes with persistent centre-involved DMO following at least 6 months of anti-VEGF therapy [41], provides an initial guideline for when one might consider changing therapy, and represents an important step in this direction.

### Summary

#### What was known before

- There is marked inter-subject variability in anatomical and functional responses to anti-VEGF therapy in DMO.
- Indirect evidence from the RISE and RIDE trials suggests that persistence of macular oedema during anti-VEGF therapy might limit subsequent vision gain.
- Post hoc analysis of Protocol I study data failed to show an association between the reduction in macular thickness at week 12 and long-term vision improvement in ranibizumab-treated eyes with centre-involved DMO.

#### What this study adds

- Based on OCT assessments over multiple time points, this analysis of Protocol I study data demonstrates that residual oedema exposure (intensity and duration) over the first 12 months of treatment is a negative prognostic factor for long-term vision outcome in ranibizumab-treated eyes with centre-involved DMO.
